# Anatomical variations of hepatic arteries: implications for clinical practice

**DOI:** 10.3389/fsurg.2025.1593800

**Published:** 2025-06-18

**Authors:** Austeja Samuolyte, Raminta Luksaite-Lukste, Mindaugas Kvietkauskas

**Affiliations:** ^1^ Clinic of Family Medicine, Faculty of Medicine, Vilnius University, Vilnius, Lithuania; ^2^ Department of Radiology, Nuclear Medicine and Medical Physics, Institute of Biomedical Sciences, Faculty of Medicine, Vilnius University, Vilnius, Lithuania; ^3^ Clinic of Gastroenterology, Nephrourology, and Surgery, Institute of Clinical Medicine, Faculty of Medicine, Vilnius University, Vilnius, Lithuania

**Keywords:** hepatic arterial variations, hepatic vasculature, hepatopancreatobiliary surgery, interventional radiology, preoperative imaging

## Abstract

Variations in hepatic arterial anatomy are highly prevalent, occurring in 45% to 75% of individuals. Understanding hepatic arterial anatomy is critical in optimizing surgical outcomes in hepatopancreatobiliary surgery, transplantation, and interventional radiology. Aberrant arteries are prone to accidental injury during surgery, which may lead to life threatening complications, such as hemorrhage or ischemia. Therefore, a clear understanding of hepatic arterial anatomy is essential for reducing intraoperative risk and improving surgical outcomes. To address this need, the present review provides an overview of hepatic arterial variants according to Michel's classification, aiming to enhance anatomical awareness and contribute to improved procedural planning across disciplines.

## Introduction

Atypical hepatic arterial variants are observed in approximately 20%–45% of individuals, with some surgical cohorts reporting even higher rates of up to 60% (1–7). These variations are clinically relevant in hepatopancreatobiliary surgery, transplantation, and interventional radiology, where, if unrecognized, arterial anomalies increase the risk of complications (8–11). Visualizing hepatic arterial anatomy is critical for planning interventions involving the liver and surrounding structures (9). High-quality imaging not only facilitates precise surgical navigation but is also integral in minimizing the risk of post-operative complications (1, 3, 10, 12, 13). Despite the advanced imaging possibilities, preoperative scans may miss up to one-third of hepatic arterial variants, which remain unidentified until surgery, leaving patients at risk for hemorrhage, ischemia, or incomplete tumor resection (5, 8, 9, 11, 14). This highlights the need for accurate mapping and informed surgical planning to reduce preventable morbidity.

While several classification systems have been proposed to categorize hepatic arterial variants, Michel's classification is the most widely recognized system, offering a structured description of the 10 most common anatomical variants (6). In this mini-review, we describe hepatic arterial variants according to Michel's classification, emphasizing key anatomical features and clinical relevance.

### Normal hepatic arterial anatomy

**Figure 1:**
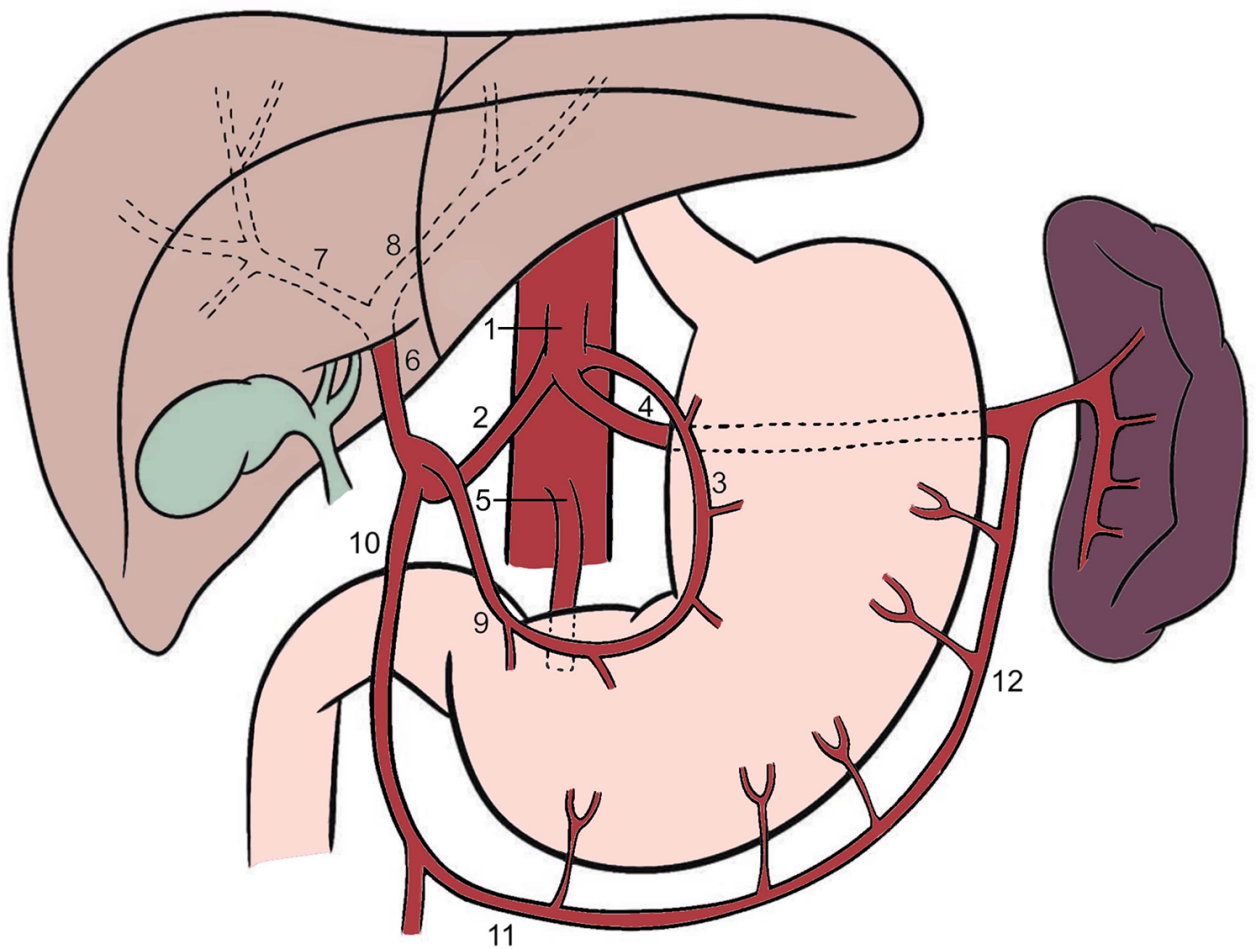
Classical anatomy of the celiac trunk and hepatic arteries. 1—celiac trunk, 2—common hepatic artery, 3—left gastric artery, 4—splenic artery, 5—superior mesenteric artery, 6—proper hepatic artery, 7—right hepatic artery, 8—left hepatic artery, 9—right gastric artery, 10—gastroduodenal artery, 11—right gastroepiploic artery, 12—left gastroepiploic artery.

In a classical hepatic arterial anatomy (Figure 1), blood reaches the liver through the two main vessels: the portal vein carrying nutrient-rich blood, and the hepatic artery which carries oxygenated blood (15). The common hepatic artery [CHA (2), *a. hepatica communis*] arises from the celiac trunk [CT (1); *truncus celiacus*]. The CT also gives rise to the left gastric artery [LGA (3); *a. gastrica sinistra*] and the splenic artery [SA (4); *a. lienalis*]. The CHA, LGA and SA form a trifurcation and are sometimes still referred to as *Tripus Halleri*, first described by the Swiss anatomist and physiologist *von Haller* (12). The LGA divides into anterior and posterior branches, which course toward the lesser curvature of the stomach, where they form anastomosis with the right gastric artery [RGA (9); *a. gastrica dextra*], arising from the CHA (3). After originating from the CT, the CHA travels along the superior edge of the pancreas, travelling through the Winslow's canal, also known as epiploic foramen. Then it bifurcates, forming a “Y” shape, giving rise to the proper hepatic artery [PHA (6); *a. hepatica propria*] and the gastroduodenal artery [GDA (10); *a. gastroduodenalis*] (8, 9). The PHA divides into anterior and posterior branches, which supply the anterior and posterior parts of the right liver lobe, respectively. The LHA [8] also gives rise to smaller vessels which supply segments II, III, and IV of the liver. The caudate lobe is vascularized by the branches that arise from both the left and right hepatic arteries. Segment IV of the liver is supplied by the middle hepatic artery (MHA; *a. hepatica media*) which always arises from the CHA, either directly or indirectly (12).

### Comparative overview of hepatic artery classification systems

Several classification systems have been created to describe hepatic arterial anatomy (Table 1). The most significant contribution was the now standard classification system published in 1966 by American anatomist Neil A. Michel (Table 2). Based on the analysis of 200 cadaver dissections, it describes the 10 most common arterial variants. Although the sample size may be considered moderate, the classification remains widely used in academic and clinical settings because of its anatomical clarity and surgical relevance (6). In 1994 Hiatt et al. proposed a simplified classification based on 1,000 liver transplant donor cases. Hiatt system presented 6 categories instead of 10, which made the system easier to apply clinically, though at the cost of reduced anatomical detail. While easier to use, it did not capture the complexity of arterial branching, and thus, offered limited insight for surgical planning (7).

**Table 1:** Summary of hepatic arterial classification systems.

Classification system	Year	Basis of study	Number of types	Notable features	Limitations
Michel's (6)	1966	Cadaveric dissection (200 cases)	10	First detailed categorization of replaced and accessory arteries	Does not encompass all rare variations
Hiatt's (7)	1994	Liver transplant donors (1,000 cases)	8	Simplified for surgical application	May overlook rare variants
Sureka's (17)	2013	Imaging studies MDCT angiography (600 cases)	Varies	Incorporates CHA origin variations; adapted for radiologic planning	Less standardized terminology
Song's (16)	2010	Pediatric CT/angiography imaging studies (5,002 cases)	Varies	Focuses on celiac trunk and hepatic artery variants in children	Limited to pediatric population

**Table 2:** Hepatic arterial anatomy variations according to michel‘s classification.

Type	Illustration		Description	Prevalence (%)
I	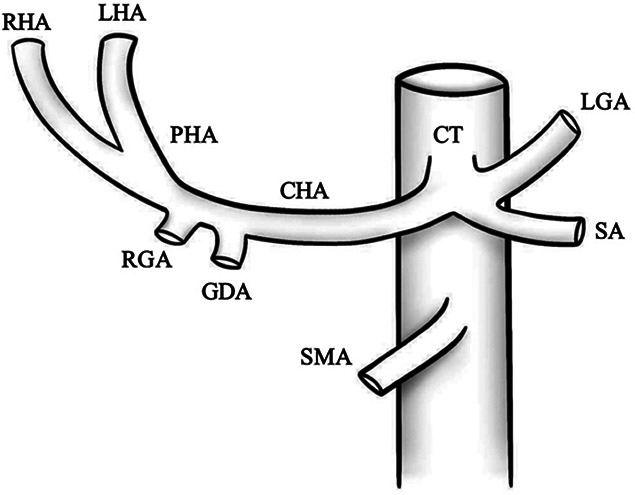		Type I—normal and most common hepatic anatomy. Common hepatic artery (CHA), arises from the celiac trunk (CT) followed by the proper hepatic artery and splits into the right hepatic artery (RHA) and left hepatic artery (LHA) via the proper hepatic artery (PHA)	55–80.91% (2, 12, 40)
II	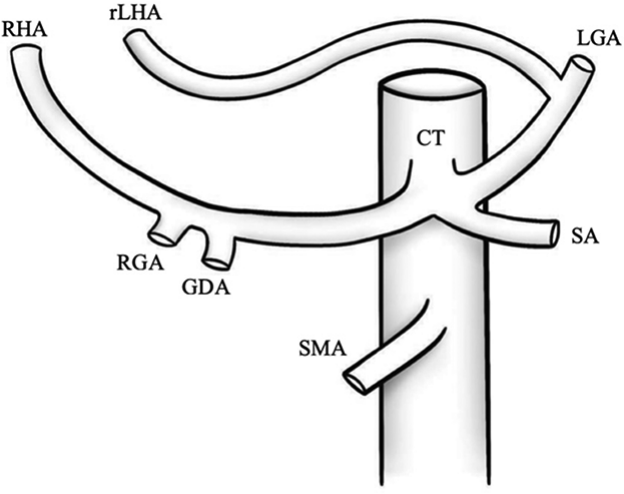		Type II—left hepatic artery (LHA) arises from left gastric artery (LGA)	0.36–10.0% (12, 20)
III	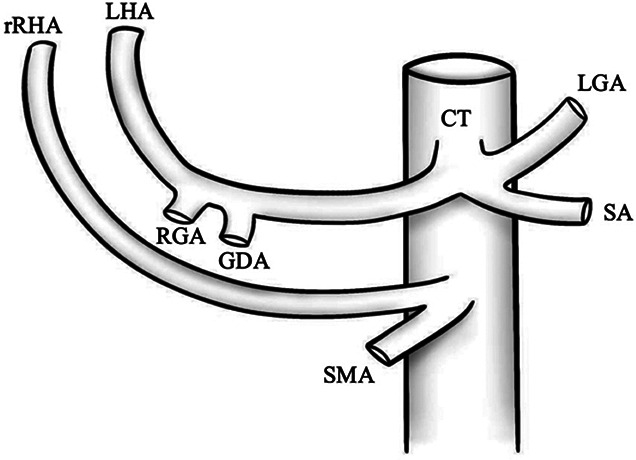		Type III—right hepatic artery (RHA) arises from superior mesenteric artery (SMA)	8.7–11.0% (12)
IV	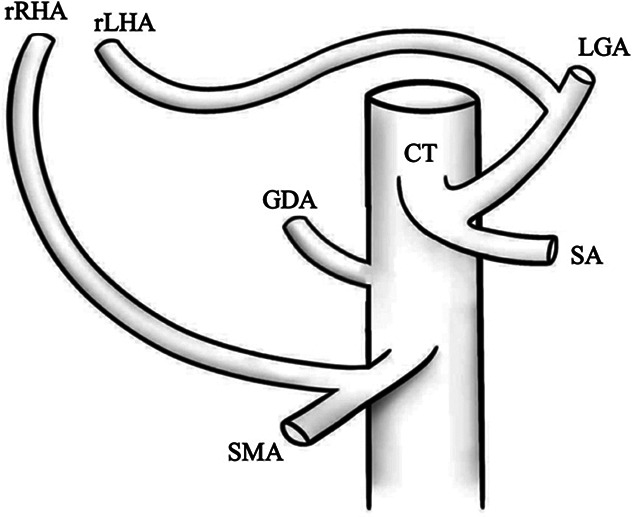		Type IV—left hepatic artery (LHA) arises from left gastric artery (LGA) and right hepatic artery (RHA) originates from superior mesenteric artery (SMA)	<1.0–11.0% (9, 12)
V	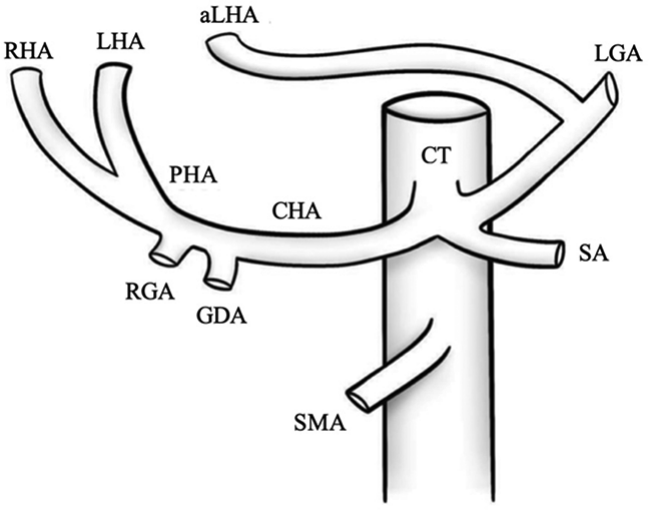		Type V—accessory left hepatic artery (aLHA) arises from left gastric artery (LGA)	1.0–8.0% (9, 12)
VI	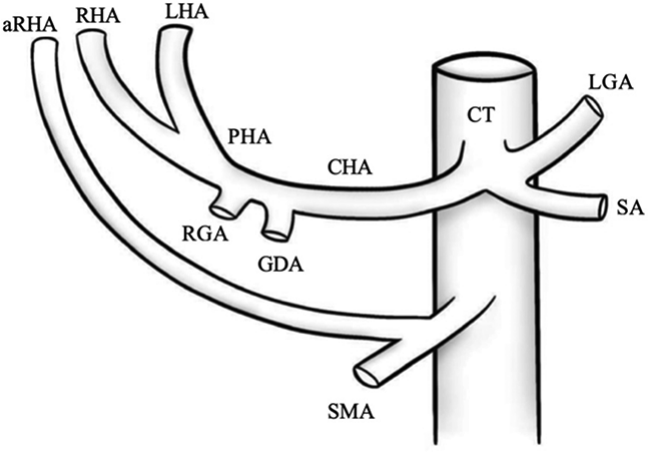		Type VI—accessory right hepatic artery (aRHA) arises from superior mesenteric artery (SMA)	7.0–10.7% (9, 12)
VII	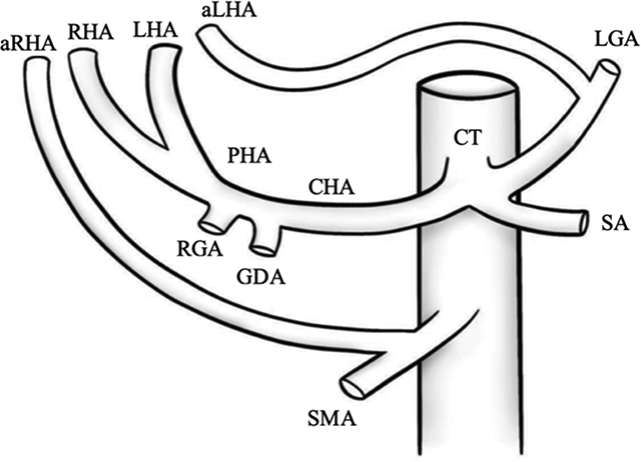		Type VII—accessory left hepatic artery (aLHA) arises from left gastric artery (LGA) and accessory right hepatic artery (aRHA) originates from superior mesenteric artery (SMA)	6.62% (20)
VIII	A	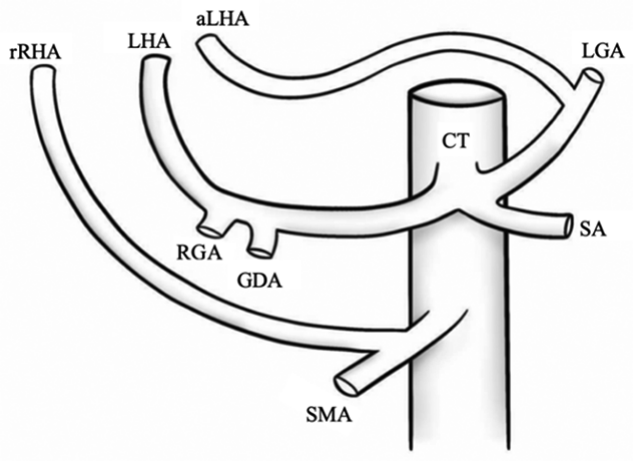	Type VIII (A)—replaced right hepatic artery (rRHA) arises from superior mesenteric artery (SMA) and accessory left hepatic artery (aLHA) originates from left gastric artery (LGA)	2.0–4.0% (12)
	B	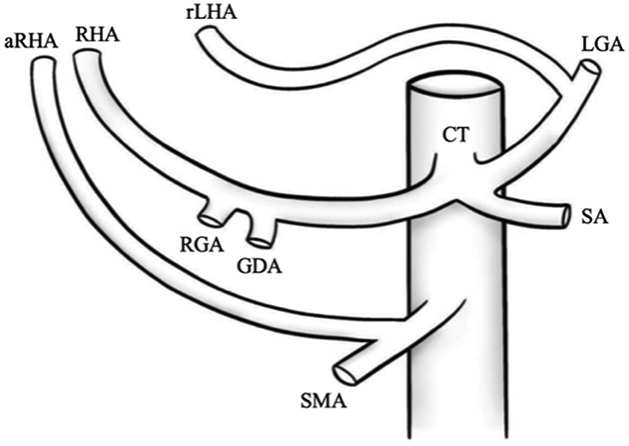	Type VIII (B)—replaced left hepatic artery (rLHA) arises from left gastric artery (LGA) and accessory right hepatic artery (aRHA) originates from superior mesenteric artery (SMA)	
IX	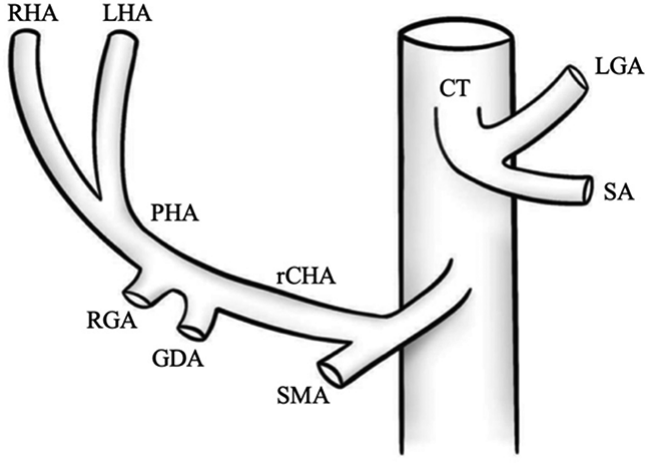		Type IX—the replaced common hepatic artery (rCHA) originates from the superior mesenteric artery (SMA)	1.0–4.5% (12)
X	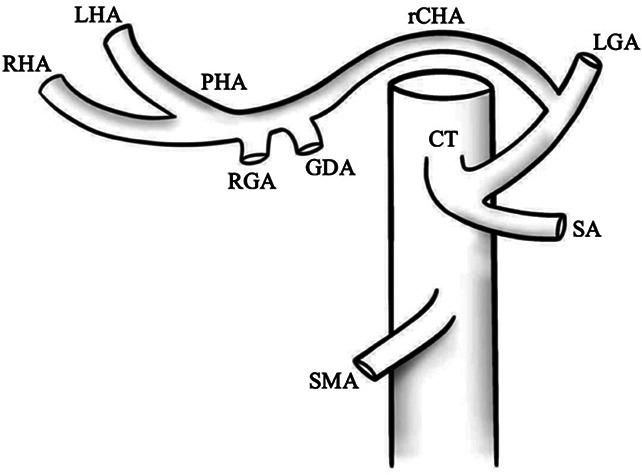		Type X—the replaced common hepatic artery (rCHA) originates from the left gastric artery (LGA)	0.5% (12)

CHA, common hepatic artery; CT, celiac trunk; PHA, proper hepatic artery; RHA, right hepatic artery; LHA , left hepatic artery; LGA, left gastric artery; SMA, superior mesenteric artery; SA, splenic artery; RGA, right gastric artery; GDA, gastroduodenal artery; aLHA, accessory left hepatic artery; aRHA, accessory right hepatic artery; rRHA, replaced right hepatic artery; rLHA, replaced left hepatic artery; rCHA, replaced common hepatic artery.

More recent imaging-based systems were proposed by Song et al. and Sureka et al. in 2010 and 2013, respectively. Song's system focuses on pediatric imaging. It highlights developmental anomalies, thus its use is mostly restricted to the pediatric population. Song's system provides insight into early vascular development, which aids in diagnosing and treating pediatric hepatobiliary conditions; however, it has limited applicability in adult surgical practice (16). Sureka's classification is based on multidetector CT angiography and is specifically designed for preoperative evaluation. It incorporates common hepatic artery variations and complements Michel's anatomical framework by including additional variants involving the origin of the common hepatic artery (CHA), which Michel's system does not fully adress (17, 18).

### Variations of hepatic arterial anatomy according to Michel’s classification

According to Michel's classification, more than half of the population has one of the atypical hepatic arterial variants (12, 19). Within this classical framework, three key terms are used in the classification to describe arterial positioning:

Accessory artery provides additional blood supply for the tissues, together with the already existing arteries of a regular anatomic course (6, 20).

Replaced artery is a vessel that originates from a location different from the usual anatomical position. In the context of hepatic circulation, a replaced artery supplies blood to the liver segments that would normally be served by standard artery, which is absent in this case.

Aberrant artery is an umbrella term for accessory and replaced arteries. In short, an aberrant artery is one that, compared to the normal anatomical variant, branches atypically. This term indicates that the course of the artery is altered, but it does not specify whether the artery provides additional blood flow alongside other arteries supplying the same area or if it is the only artery providing blood supply.

### Imaging of hepatic arterial variations

Several imaging modalities are available for evaluating hepatic arterial anatomy, namely computed tomography angiography (CTA), magnetic resonance angiography (MRA), and digital subtraction angiography (DSA). The first choice for imaging is CTA, which effectively visualizes arterial branching, stenosis, and occlusions (19, 21). The main advantage of CTA is its ability to obtain high-quality imaging data of a broad anatomical region. Moreover, it offers shorter acquisition time compared to MRA (22). Three-dimensional vascular CTA reconstructions are particularly valuable for preoperative assessment of arterial branching and spatial relationships with adjacent structures. CTA enables precise visualization of small-calibre and short arterial segments, which is achieved through maximum intensity projection and automated volume rendering techniques (3).

Alternative imaging modalities, such as MRA and DSA, have limited clinical applicability regarding hepatic arterial anatomy visualization. Although MRA does not involve ionizing radiation, it requires longer imaging time and offers lower spatial resolution. MRA is mainly indicated for the assessment of aneurysms, dissection, coarctation, and arterial anomalies, and therefore, has limited relevance in mapping hepatic arterial anatomy (23, 24). DSA is primarily reserved for therapeutic interventions (thrombectomies, stenting, embolization) rather than routine arterial imaging because of its invasive nature (25–27).

### Clinical significance of variations in hepatic arterial anatomy

A detailed assessment of hepatic arterial anatomy plays a pivotal role in the preoperative phase of liver surgeries, particularly resections and transplantations, which are among the most common surgeries requiring arterial imaging (28, 29). Research has demonstrated that atypical arterial anatomy is associated with an increased incidence of hepatic artery thrombosis (HAT) in liver transplantation (30). A study analyzing 836 liver transplantations reported that patients with arterial reconstructions due to variant anatomy had a higher risk of developing early HAT, with arterial reconstruction identified as an independent risk factor [adjusted hazard ratio (aHR) = 3.72] (31).

Identifying aberrant arteries is pivotal in oncologic hepatobiliary surgeries, especially in pancreaticoduodenectomy (the Whipple procedure). One of the variants particularly vulnerable to injuries is Michel's type III, where a replaced RHA arises from the SMA and often courses through or close to the pancreatic head, making it susceptible to intraoperative injury and potentially compromising oncologic resection margins (32, 33). Moreover, a 2022 study of 207 patients undergoing pancreatic head resection confirmed the presence of an accessory RHA as an independent predictor of hepatic recurrence, with 61.8% in patients with an aberrant RHA vs. 25.3% of those without an atypical arterial variant (*P* < 0.0001) (34). Michel's type II (replaced LHA from LGA) variation poses a risk of left hepatic lobe ischemia in distal pancreatectomy with celiac axis ligation, as the arterial supply to the left liver lobe may be interrupted by inadvertent ligation of the LGA. Michel's type IX (replaced CHA from SMA) places the liver and bile duct at risk of ischemia as dissection near SMA may damage a single arterial source that is responsible for multiple organ blood supply (35).

During laparoscopic cholecystectomy, RHA is the most commonly injured vessel (21, 36, 37). Michel's type III (replaced RHA from SMA), may increase the risk of intraoperative bleeding and bile duct injury. Extensive knowledge of anatomical variations improves dissection and minimizes the likelihood of adverse surgical events, especially while preoperative arterial imaging is not usually performed before cholecystectomy (21). For example, if surgeons are not aware of RHA variations, such as in the case of unclear Calot's triangle, RHA could be accidentally injured or mistaken for cystic artery and cut off (21). Recent evidence confirms that hepatic arterial variations (like a replaced or accessory RHA) are clinically significant risk factors for complications in cholecystectomy, for example intraoperative hemorrhage occurred in 16 out of 95 patients with anatomical variants (16.8%) vs. 4 out of 203 in the normal group (1.9%) (*P* < 0.001) ( (38).

In interventional radiology, radioembolization presents a particular challenge, as inaccurately assessed aberrant arteries can compromise procedural effectiveness and lead to suboptimal distribution of radioactive microspheres (4). In chemoembolization and intra-arterial liver chemotherapy, poor knowledge of the anatomical variations may lead to ineffective treatment or potential damage to the hepatic parenchyma or the adjacent soft tissue structures (9). For example, navigating Michel's type III (replaced RHA from SMA), may require an altered catheterization strategy due to its retroportal course, which results in more complicated catheter access (39). Therefore, meticulous vascular mapping should be considered standard practice in interventional hepatic procedures involving arterial access.

## Conclusions

Anatomical variations of hepatic arteries are highly prevalent and clinically significant. Detailed understanding and accurate reporting of these variants are essential for reducing procedural risks in hepatopancreatobiliary surgery, liver transplantation, and interventional radiology. Increased awareness of hepatic arterial variants should be encouraged in clinical practice and education to ensure more effective patient care.
